# Rib fracture detection system based on deep learning

**DOI:** 10.1038/s41598-021-03002-7

**Published:** 2021-12-06

**Authors:** Liding Yao, Xiaojun Guan, Xiaowei Song, Yanbin Tan, Chun Wang, Chaohui Jin, Ming Chen, Huogen Wang, Minming Zhang

**Affiliations:** 1 Department of Radiology, The Second Affiliated Hospital, Zhejiang University School of Medicine, No.88 Jiefang Road, Shangcheng District, Hangzhou, 310009 Zhejiang China; 2 Hithink RoyalFlush Information Network Co., Ltd, No. 18 Tongshun Street, Yuhang District, Hangzhou, 310012 Zhejiang China

**Keywords:** Diseases, Health care, Medical research

## Abstract

Rib fracture detection is time-consuming and demanding work for radiologists. This study aimed to introduce a novel rib fracture detection system based on deep learning which can help radiologists to diagnose rib fractures in chest computer tomography (CT) images conveniently and accurately. A total of 1707 patients were included in this study from a single center. We developed a novel rib fracture detection system on chest CT using a three-step algorithm. According to the examination time, 1507, 100 and 100 patients were allocated to the training set, the validation set and the testing set, respectively. Free Response ROC analysis was performed to evaluate the sensitivity and false positivity of the deep learning algorithm. Precision, recall, F1-score, negative predictive value (NPV) and detection and diagnosis were selected as evaluation metrics to compare the diagnostic efficiency of this system with radiologists. The radiologist-only study was used as a benchmark and the radiologist-model collaboration study was evaluated to assess the model’s clinical applicability. A total of 50,170,399 blocks (fracture blocks, 91,574; normal blocks, 50,078,825) were labelled for training. The F1-score of the Rib Fracture Detection System was 0.890 and the precision, recall and NPV values were 0.869, 0.913 and 0.969, respectively. By interacting with this detection system, the F1-score of the junior and the experienced radiologists had improved from 0.796 to 0.925 and 0.889 to 0.970, respectively; the recall scores had increased from 0.693 to 0.920 and 0.853 to 0.972, respectively. On average, the diagnosis time of radiologist assisted with this detection system was reduced by 65.3 s. The constructed Rib Fracture Detection System has a comparable performance with the experienced radiologist and is readily available to automatically detect rib fracture in the clinical setting with high efficacy, which could reduce diagnosis time and radiologists’ workload in the clinical practice.

## Introduction

Rib fractures are the most common consequences of traumatic chest injury^1^, most commonly caused by motor vehicle accidents, sport, falls etc. A recent study that included 4,168 patients with thoracic trauma in East China showed that 66.8% of the patients sustained rib fractures^2^. Rib fracture is also an important indicator of trauma severity, and patients with rib fractures have a higher admission rate and mortality than those without^3^. Many studies have highlighted that there is a high morbidity and mortality rate with even a single rib fracture, and as the number of rib fractures increases, patient’s morbidity and mortality rates increase^1,4^. In addition, accurate detection of rib fracture can have medical-legal relevance^5^. Therefore, it is essential to accurately diagnose the location and the number of rib fractures in the clinical setting.

Plain X-ray and computed tomography (CT) are the most common imaging modalities used for rib fracture detection. In comparison to CT, the plain X-ray is convenient and fast, but the detection rate is relatively poor with more than 50% of rib fractures missed^1,6^. CT is the main imaging modality used to evaluate thoracic trauma for rib fractures and associated complications^1^. With chest CT, a large number of imaging sections and series are generated which consist of 12 pairs of ribs with heterogeneous shapes. All images must be evaluated sequentially, rib-by-rib and side-by-side, which is time-consuming and demanding^5^. Despite the best human effort, a misdiagnosis rate between 19.2 to 26.8% was reported with chest CT for rib fractures^7,8^, some of which may potentially lead to serious consequences^9^. Therefore, it is essential to develop an assistant machine learning detection system for rib fractures to minimize misdiagnosis.

Deep learning allows raw data to be fed into computer models and automatically processed with multiple pattern extraction and weighting levels^10^. Deep learning has been widely acknowledged for its great potential in complex pattern recognition and learning images with spatial hierarchy in multiple medical fields such as dermatology, radiology, ophthalmology, and pathology^11^, as well as in various fracture detections such as radial and ulnar fracture^12^, wrist fracture^13^, and thoracolumbar fracture etc.^14^. Therefore, it would also be feasible to construct a rib fracture detection system based on a deep learning model. Recently, Zhou et al.^15^, Weikert et al.^16^ and Jin et al.^17^ constructed several deep learning models for rib fracture detection with a high diagnostic sensitivity and specificity, in particular, such models have the advantage of dramatically reducing the diagnosis time. Since the performance of a deep learning model is highly dependent on the characteristics of the trained data, such as imaging resolution and fracture extent, the model’s generalizability remains a constant issue. In general, existing deep learning models often require localized data input and refinement for validation before clinical translation.

From the deep learning perspective, rib fracture diagnosis is defined as an object detection problem, and a number of methods, for example, Faster-RCNN^18^, SSD^19^, YOLO-v3^20^, have been previously proposed. Because the rib fracture region is relatively small and imperceptible within many chest CT images, such detection system is more difficult compared to other fracture detection systems. Therefore, in order to construct a deep-learning model specific for rib fracture, this study had developed a three-step method to detect rib fracture in a patient population with mild to severe rib fractures: (1) a semantic segmentation model was trained and used to extract all bony features from chest CT; (2) a rib location model was trained to extract ribs and remove vertebrae, scapulae and sternum; (3) a classification model was trained to identify the fracture in the extracted ribs, and the performance was further tested using an independent dataset. Finally, the performance of radiologist-model collaboration was evaluated after the completion of deep-learning model.

## Materials and methods

### Data collecting

This study was approved by the Ethics Committee of the Second Affiliated Hospital of Zhejiang University with the informed consent waived. All the methods were carried out in accordance with relevant guidelines and regulations. A total of 1707 patients from the Second Affiliated Hospital of Zhejiang University were enrolled in this study. All chest CT scans were collected using keyword searches in Picture Archiving and Communication Systems (PACS) between January 1, 2016 and March 31, 2019. The inclusion criteria were: (a) slice thickness after image reconstruction less than 2 mm, (b) at least 1 rib fracture present, (c) no significant artifact in the images. Finally, a total of 1707 patients with chest CT images were included in this study. To ensure that all data was analyzed at the same windowing level, the window center and window width parameters of all CT scans were set to 400 and 1600, respectively.

### Data annotation and pretreatment

All CT images were annotated by three experienced radiologists (all with over 10 years of experience in CT diagnosis) and checked by two senior radiologists (both with more than 15 years of experience in CT diagnosis) as the ground truth for both the training, validation and testing sets of rib fracture detection model. In the situation where the annotation result of data was inconsistent, all five radiologists were invited to participate in a discussion, and a consensus decision was made. All CT images were annotated on a medical image processing and navigation software, 3D Slicer^21^, by drawing a mask around the bone region and a rectangular bounding box surrounding the rib fracture.

**Figure 1:**
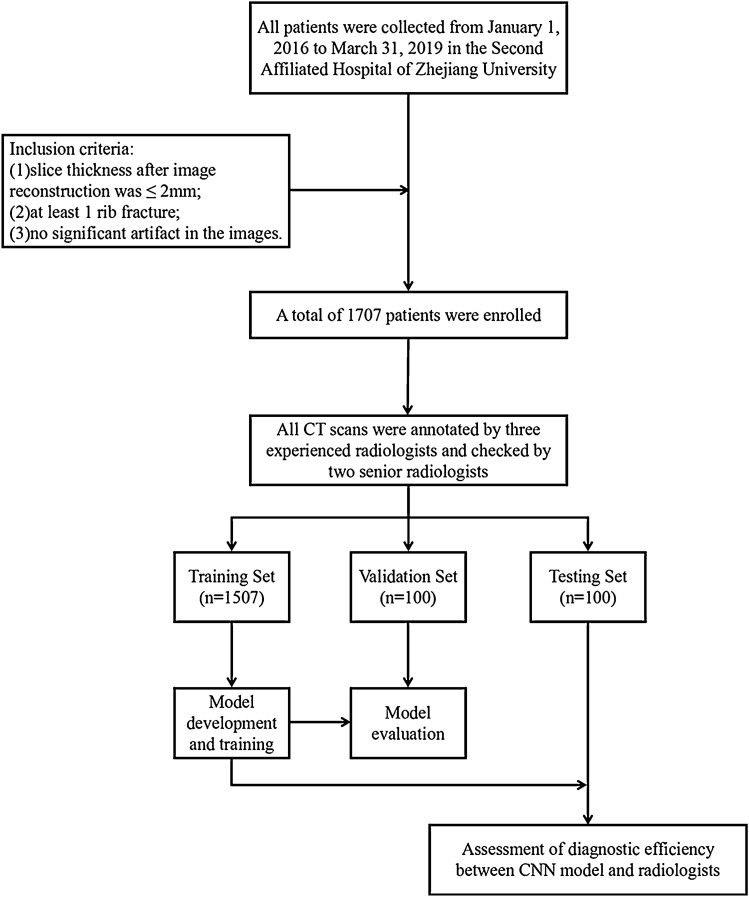
Flow chart showing overall study process.

As shown in Fig. 1, patients were allocated into the training set (1507 cases, 581,701 slices, and 7362 fractures), the validation set (100 cases, 36,697 slices, and 473 fractures) and the testing set (100 cases, 37,183 slices, and 436 fractures) according to the examination time. The deep learning model for rib fracture detection was developed and trained on the training set. The hyper-parameters of the deep learning system were finetuned using the validation set. The diagnostic efficiency of the deep learning model was assessed on the testing dataset, together with the evaluation of the efficacy of the deep-learning model alone, radiologist alone and radiologist-model collaboration. These 100 cases for testing were composed of 62 male and 38 female subjects with a mean age of 56.7 $\pm$ 12.8 years (range from 23–88 years). The overview of dataset was descripted in Table 1.

**Table 1:** The overview of dataset.

Cohorts	No. Patients	No. CT slices	No. Fractures
Training	1507	581,701	7362
Validation	100	36,697	473
Testing	100	37,183	436

### Model construction

**Figure 2:**
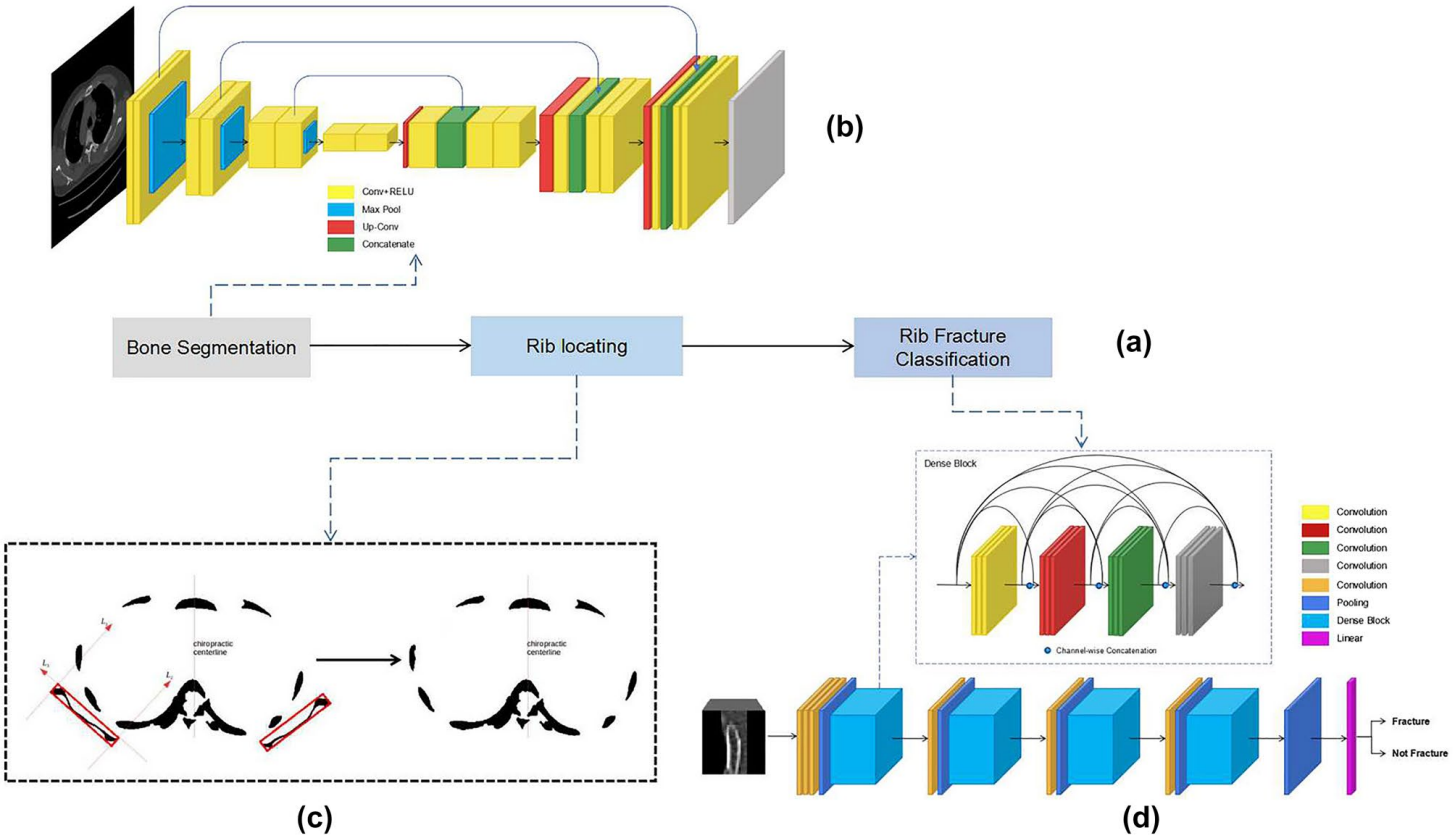
The pipeline for detecting rib fractures from CT scans. The fracture detection task was divided into three stages, including bone segmentation, rib location, and rib fracture classification (**a**). (**b**) bone segmentation; (**c**) rib location; (**d**) rib fracture classification.

The extents of rib fractures were highly varied and sometimes only a few abnormal pixels can be observed in very small fractures, which make them more difficult to detect. As illustrated in Fig. 2, we divided the fracture detection model into three-step algorithms, including bone segmentation, rib location, and rib fracture classification.

- Bone segmentation: U-Net^22^ was implemented and trained for bone segmentation. U-Net is an encoder-decoder architecture. The encoder was a typical Convolutional Neural Network (CNN) applied to extract semantic features from basic patterns to complex spatial patterns. In the decoder, a feature map resolution was gradually restored through a series of transposed convolution operations. Features from the encoder were reprocessed through feature cascade from levels of the encoder and decoder.
- Rib location: Because ribs were difficult to distinguish from scapulae, sternum, and vertebrae when performing bone segmentation, we removed vertebrae and scapulae based on their characteristic shapes and locations as a critical preprocessing step.
- Rib fracture classification: 3D DenseNet was employed for rib fracture classification, which was an extension of DenseNet^23^ by extending 2D Convolution and 2D pooling into 3D. It connected each layer to every other layer in a feed-forward fashion, which alleviated the problem of gradient dissipation, facilitated the propagation and the reuse of features. An inception structure^24^ was introduced to make the network learn features in different scales of receptive fields. Focal loss function^25^ was calculated as the loss function during training, which enhanced the generalization ability of the model by minimizing the difference between the ground truth and training outcome, especially on hard cases.

The details of the procedures mentioned above were described in the Supplementary Materials.

### Model training

- The training of U-Net: U-Net was trained on a training set consisting of 4496 rib CT images. An input image of size $512 \times 512$ was randomly cropped from a CT image. During training, each batch contained 8 samples. Data augmentation, such as flipping, contrast adjustment and random noise, was manipulated to avoid overfitting^26^. The optimization objective function was computed by the dice-loss^27^ function, and the calculation formula of the dice loss function was:

$${\text{L}}_{\text{dice}} = 1 - \frac{2\mathop\sum\nolimits_{{{\text{i}} = 1}}^{{{\rm N}}}{\text{p}}_{{\text{i}}}*{\text{g}}_{{\text{i}}} + {\upepsilon}}{\mathop \sum\nolimits_{{{\text{i}} = 1}}^{{{\rm N}}}{\text{p}}_{\text{i}} +\mathop \sum \nolimits_{{\text{i}} =1}^{{\rm N}} {\text{g}}_{\text{i}} + {\upepsilon }}$$

where ${\text{N}}$ was the number of pixels in an input image, ${\text{p}}_{{\text{i}}}$ was the predicted value, and ${\text{g}}_{{\text{i}}}$ was the ground truth. A small constant ε was also inserted to avoid the situation where the denominator was zero. The U-Net was trained using Adam optimizer with a warm-up strategy. The learning rate was set to 0.01 and then linearly decreased to 0.001 in 100 epochs.

(b) The training of 3D DenseNet: A total of 91,574 fracture blocks and 50,078,825 normal blocks were extracted from the training set. One case contained a series of images which could be regarded as ${\text{H}} \times {\text{W}} \times {\text{D}}$ volume data. Let ${\text{h}}$ and ${\text{w}}$ be the height and width of minimum circumscribed rectangles of all ribs on each image in the series, and a $\left( {{\text{h}} + 6} \right) \times \left( {{\text{w}} + 6} \right) \times 5$ 3D data block was sampled, and then the block was normalized into $48 \times 48 \times 5$ in non-deformation way. To normalize the 3D data block in non-deformation way, a minimum circumscribed square of each 3D data block was firstly drawn. Then, the margin in the square was padded with pure black pixels to generate a new 3D data block. Finally, the new 3D data block was resized into $48 \times 48 \times 5$. During training, we adopted a sampling strategy to alleviate the imbalance between positive and negative samples by allowing each batch to contain 4 positive samples and 4 negative samples. Data augmentation, such as flipping, contrast adjustment and random noise, was applied to avoid overfitting. The optimization objective function was computed by focal loss function. The 3D DenseNet was further trained again using Adam optimizer with a warm-up strategy and the learning rate was initially set to 0.01 then decreased to 0.001 in 100 epochs.

### Model evaluation and statistical analysis

- Bone Segmentation: for bone segmentation model, the average IOU (intersection over union) and the Dice coefficient^28^ were used for the evaluation of U-Net. IOU is a good metric for measuring overlap between two masks, and it can be defined as follows:

$${\text{IOU}} = \frac{{\left| {{\text{A}} \cap {\text{B}}} \right|}}{{\left| {{\text{A}} \cup {\text{B}}} \right|}}$$

The Dice Similarity Coefficient (DSC) is a statistic used to evaluate the similarity of two masks. The DSC is defined as

$${\text{DSC}} = \frac{{2\left| {{\text{A}} \cap {\text{B}}} \right|}}{{\left| {\text{A}} \right| + \left| {\text{B}} \right|}}$$

where A is a set of rib regions annotated by the radiologists, and B is a set of segmented rib regions by the algorithm.

(b) Rib fracture classification: to compare the efficiency of the trained deep learning model with that of radiologists in diagnosing rib fracture, we proposed three testing groups in our study, including the deep learning model alone, radiologist alone, and radiologist-model collaboration. Two radiologists (radiologist A with 3 years of experience in CT diagnosis; radiologist B with 8 years of experience in CT diagnosis) who were blind to patient information and imaging annotation were invited to participate in the study. Both radiologists were informed of the gold standard criteria for rib fracture classification before the test, and their detected fracture location and diagnosis time were recorded. After three months, both radiologists were invited to diagnose the same set of chest CT images with the assistance of the constructed detection model and same parameters were taken. Two sample t-test was calculated to compare the detection or diagnosis time required for deep learning model alone, radiologist alone, and radiologist-model collaboration.

Precision (positive predictive value, PPV)^29^, recall^29^, F1-score^30^ and negative predictive value (NPV)^29^ were selected as evaluation metrics for three testing groups. Precision was defined as the ratio of correctly predicted positive observations to the total predicted positive observations. Recall was the ratio of correctly predicted positive observations to the all observations in actual class. F1- score was the weighted average of Precision and Recall. NPV was the ratio of correctly predicted negative observations to the total predicted negative observations. These metrics can be formulated as follows:

$$\begin{aligned} {\text{precision}} & = \frac{{{\text{TP}}}}{{{\text{TP}} + {\text{FP}}}} \\ {\text{recall}} & = \frac{{{\text{TP}}}}{{{\text{TP}} + {\text{FN}}}} \\ {\text{F}}1 - {\text{score}} & = 2 \times \frac{{{\text{precision}} \times {\text{recall}}}}{{{\text{precision}} + {\text{recall}}}} \\ {\text{NPV}} & = \frac{{{\text{TN}}}}{{{\text{TN}} + {\text{FN}}}} \\ \end{aligned}$$

where “TP” were these ribs classified by the algorithm as rib fractures and also by the radiologists, “FP” were these ribs classified by the algorithm as rib fractures but not by the radiologists, “TN” were corresponding to these ribs classified as not belonging to rib fractures both by the algorithm and the radiologists, and “FN” were these ribs classified as not belonging to rib fractures but they were corresponding to rib fractures bones according to the annotation of the radiologists.

Finally, the sensitivity and average number of the false positives (FPs) per patient were analyzed using a free-response ROC (FROC) curve^31^, because FROC was a tool for characterizing the performance at all thresholds simultaneously. The diagnosis and detection time were recorded.

## Results

Due to the heavy workload of bone annotation, only 4496, 3145 and 3568 CT images were annotated for the training, validation and testing of U-Net in bone segmentation, respectively. The average IOU in the test dataset was 0.8462, and the DSC was 0.9167. It was worth mentioning that the edge of a bone was ambiguous and difficult to label accurately, so it was very hard to objectively evaluate the performance of bone segmentation. And the bone segmentation with elongated shape tended to be associated with low segmentation metrics. Despite the low IOU, U-Net could effectively remove the factors that interfere with the classification of rib fractures.

**Figure 3:**
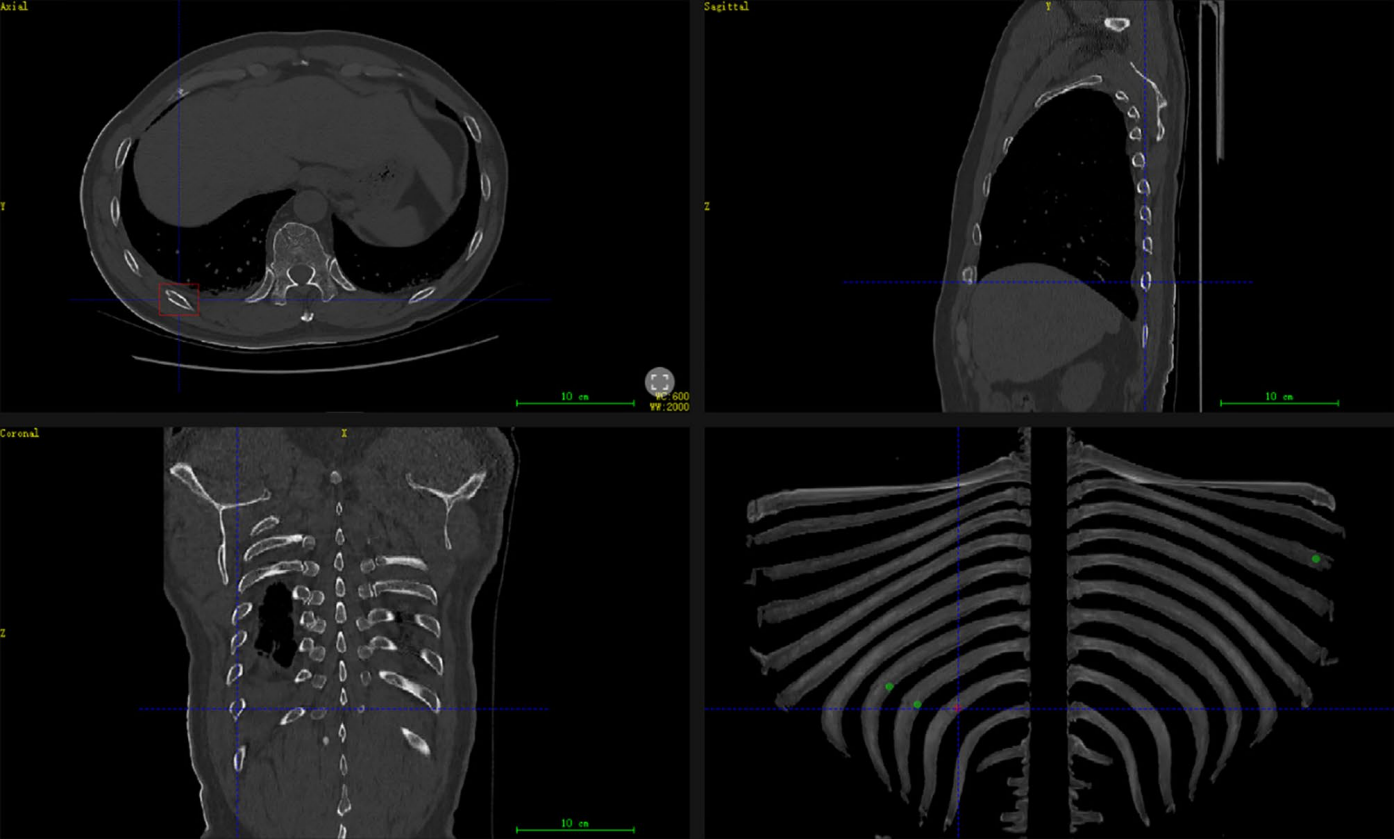
Rib 3D view. The red rectangular box was the selected fracture lesion, and the green parts were the other suspected fracture lesions detected by our Rib Fracture Detection System.

For the training of rib fracture classification, we recreated a new dataset. The overview of this dataset was shown in Table 2. For the testing of the Rib Fracture Detection System, a total of 100 cases were examined, including 436 rib fractures, with an average of 4.36 rib fractures in one case (Fractured ribs varied from 1 to 15). The result of rib fracture detection from Rib Fracture Detection System was represented in 3D view as shown in Fig. 3.

**Table 2:** The overview of dataset for the training of U-Net and 3D DenseNet.

Cohorts	U-Net	3D DenseNet	
	No. CT images	No. Fracture blocks	No. Normal blocks
Training	4496	91,574	50,078,825
Validation	3145	5981	3,323,151
Testing	3568	5992	3,452,162

**Figure 4:**
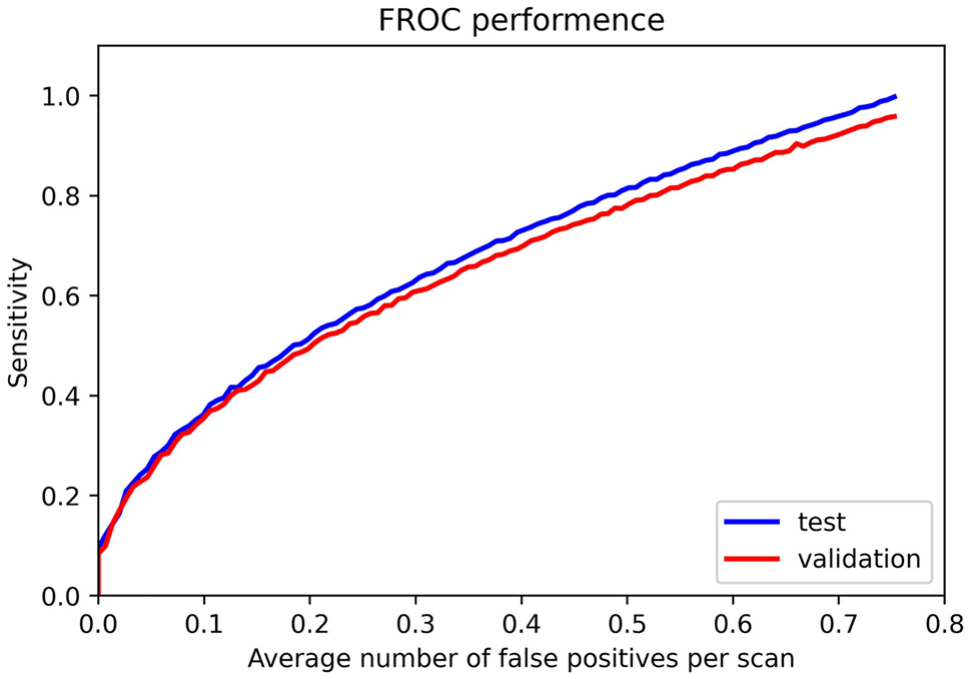
Free-response ROC (FROC) curve for our model.

To evaluate the performance of the Rib Fracture Detection System, the results of the validation set and the testing set were both reported. In the validation set, the F1- score of the Rib Fracture Detection System was 0.888 and the precision and recall were 0.864 and 0.914 respectively in the 100 cases tested with an FROC cutoff value of 0.89 (FROC curve was shown in Fig. 4). On the testing set, the F1-score of the Rib Fracture Detection System was 0.890 and the precision, recall and NPV were 0.869, 0.913 and 0.969 respectively in the 100 cases tested with the same cutoff value of the FROC threshold of 0.89.

The F1-score of radiologist A was 0.796 and the precision, recall and NPV were 0.935, 0.693 and 0.989, respectively. The F1-score of radiologist B was 0.889 and the precision, recall and NPV were 0.928, 0.853 and 0.985, respectively. Therefore, it was concluded that the Rib Fracture Detection System had a comparable performance to radiologists, but with higher recall. In addition, we tested the radiologist-model collaboration performance, and observed that, for both radiologists, their diagnostic performance were improved and their workload (diagnosis time) was significantly reduced (Table 3).

**Table 3:** The comparison of the performance between the model, radiologist and radiologist-model collaboration.

Group	Model	Radiologist A	Radiologist B	Radiologist A-model collaboration	Radiologist B-model collaboration
F1-score	0.890	0.796	0.889	0.925	0.970
Recall	0.913	0.693	0.853	0.920	0.972
Precision	0.869	0.935	0.928	0.930	0.968
NPV	0.969	0.989	0.985	0.985	0.993
Time (seconds)	20 ± 5.8	242.6 ± 83.0*	153.6 ± 34.2*	207.0 ± 47.9^a^	58.6 ± 31.4^a^

*NPV* negative predictive value.

*Indicated the p value of the comparison between model and radiologists was < 0.001.

^a^Indicated the p value of the comparison before and after using the model was < 0.001.

**Figure 5:**
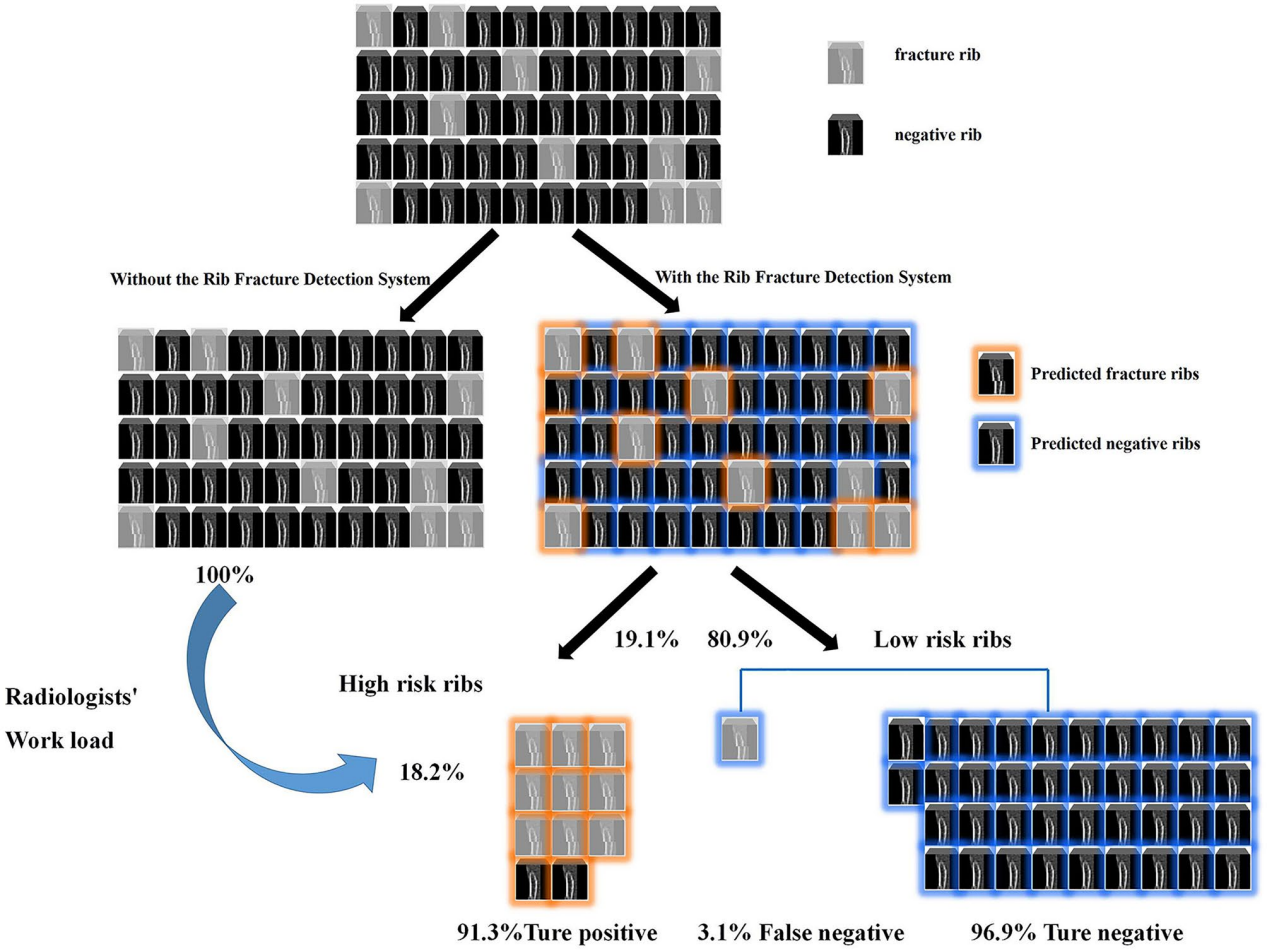
Impact of the Rib Fracture Detection System in clinical practice for patients with suspicion of rib fracture in the Department of Radiology. In a cohort of patients with suspected rib fractures who underwent chest CT investigation, radiologists should pay close attention to all of the ribs without the help of our model in order to look for 18.2% of the fractured ribs. Since 80.9% of the ribs were diagnosed as non-fracture ribs by this model with a 96.9% true-negative rate, it demonstrated high accuracy in identifying true non-fractured ribs by the constructed model. As a result, with the assistance of this deep learning model, radiologists only had to pay more attention to 19.1% of the ribs that were categorized as high-risk for fracture, which significantly reduced their workload in detecting rib fracture.

This Rib Fracture Detection System detected 458 suspected fractures and 398 true fractured ribs from these cases. An average of 4.58 suspected fractures and 3.98 ground truth annotated were detected in an average of 372 images in one case with our model, which meant if applying to the clinical setting, a radiologist only needs to review a small number of images containing suspected fractures using this model. The results demonstrated that this model had a NPV of 0.969. This emphasized the possibility of model triage, as a total of 80.9% of images were predicted as negative with 96.9% true-negatives, this left the other 19.1% images as high-risk images with a potential rib fracture. Therefore, radiologists could focus on these rib images to improve accuracy and workflow and finally reduce workload. A schematic diagram was shown in Fig. 5 to illustrate how the Rib Fracture Detection System worked and reduced the workload.

## Discussion

This study constructed a deep-learning Rib Fracture Detection System and its performance was assessed in comparison to radiologists. It was concluded that this deep-learning model had the ability to detect rib fractures with high precision and recall (precision: 0.869, recall: 0.913), which had a comparable diagnostic precision with both the junior and the experienced radiologists and was superior in term of recall ability. This indicated that this detection model would not only reduce the workload but also minimize misdiagnosis. Moreover, for both radiologists in the study, with the assistance of this detection model, their diagnostic effectiveness was significantly improved with decreased diagnosis time and increased recall.

Rib fracture detection is crucial for thoracic trauma evaluation in identifying associated injury, preventing complication, obviating potential medico legal issues, and helping patient pain management^32^. Thin-slice CT is the main imaging modality used to evaluate thoracic trauma, generating hundreds of images waiting to be analyzed. Consequently, radiologists have a high workload, and missed diagnosis becomes an inevitable issue, which is more apparent for junior radiologists^13^. To address this clinical challenge, this study suggested a Rib Fracture Detection System based on the deep learning model, which allowed to have superior recall and similar diagnostic precision to the radiologists. By using this detection system, diagnostic performance was significantly improved for both the experienced and the junior radiologists, and the workload was dramatically reduced, suggesting that such a detection system had a high potential for clinical translation.

Although several studies had recently reported the usefulness of deep-learning models in detecting rib fractures, each deep learning model required clinically suitable larger local data input with a balanced sample of mild to severe rib fractures. In some difficult mild fractures, only a few abnormal pixels could be observed, which could be easily missed. Therefore, in the present study, we performed a three-step model construction, including bone segmentation, rib location, and rib fracture classification. For rib fracture classification, 3D DenseNet was utilized as it was sensitive to even small fracture lesions with only a few abnormal pixels^23^. For rib location, the spine and scapulae removal could help eliminate the interference of the spine and scapulae. Based on our evaluation, 87 blocks in spine and scapulae were recognized as rib fractures in the rib fracture classification without the spine and scapulae removal. The sampling strategy and focal loss function were further applied to alleviate the imbalance between positive and negative samples. Focal loss is an improved version of Cross-Entropy Loss that tries to handle the imbalance between positive and negative samples by assigning more weights to hard examples. In addition, we adapted an annotation-checking-discussion workflow to ensure each sample was read by five radiologists and high accuracy of the ground truth. With these characteristics, this Rib Fracture Detection System achieved high precision and recall in rib fracture detection and improved radiologist work efficiency.

To further demonstrate the effectiveness of our proposed algorithm, we compared our algorithm with several state-of-the-art algorithms. We evaluated the performance of FracNet^17^, Fast RCNN^16^, Faster RCNN^15^, YOLOv3^15^ on the testing set of our collected dataset. The comparison between our Rib Fracture Detection System with these approaches was listed in Table 4. These results demonstrated that the performance of our Rib Fracture Detection System outperformed FracNet, Fast RCNN, Faster RCNN, and YOLOv3.

**Table 4:** The comparison of the performance between our Rib Fracture Detection System with Fast RCNN, Faster RCNN, YOLOv3.

Group	Model	Fast RCNN	Faster RCNN	YOLOv3
F1-score	0.890	0.863	0.870	0.877
Recall	0.913	0.874	0.889	0.894
Precision	0.869	0.853	0.852	0.861
NPV	0.969	0.925	0.932	0.942

Although the system performed well in most cases, this study still had several limitations. First, the deep learning model was developed and trained on CT data from a single large academic institution and the test set is relatively small, lacking multi-center or external data validation. Future research is required to determine if the same model trained can achieve high performance on larger or multi-institutional datasets. Second, this was a retrospective study and we would hope to include prospective data in the future with more radiologists as well as more chest CT cases. Third, without accurate masks for rib segmentation in the dataset, the quantitative evaluation of the precision of the spine and scapulae removal was not performed. Fourth, false positive rate is still high compared with the performance of radiologists and we hope we can reduce the false positive rate by collecting more dataset and introducing novel deep learning methods. And finally, this model could only detect rib fracture but cannot identify fracture degree and classify acute and healed rib fractures, which may be useful information to clinicians. Therefore, future studies were warranted to complete these tasks with this Rib Fracture Detection System.

## Conclusions

This study developed a Rib Fracture Detection System that achieved high performance in rib fracture detection on chest CT images based on the deep learning algorithm. By radiologist-model collaboration, radiologists can significantly reduce their workload, and minimize misdiagnosis. This Rib Fracture Detection System is readily available in the clinical setting.

## Supplementary Information

Supplementary Information.

## Data Availability

Anonymized data can be made available upon reasonable request to the corresponding author.
