# Supplementary material for: Rib fracture detection system based on deep learning
Source: Sci Rep. 2021 Dec 6;11:23513. doi: 10.1038/s41598-021-03002-7 (PMC8648839; doi:10.1038/s41598-021-03002-7)
Supplement: Supplementary file 1 — Supplementary Information. [file 41598_2021_3002_MOESM1_ESM.doc]

**Rib Fracture Detection System Based On Deep Learning（Supplementary Material）**

**Bone Segmentation**

At the first stage, we would like to get the positions of all bone. This task aims to classify each pixel from the image. Unet [1] is a classic image segmentation CNN model that has been widely used to solve medical image segmentation problems such as liver segmentation, vessel segmentation, etc, it’s a kind of “fully convolutional network [2]”, The whole network presents a U-shaped structure. For an input image, convolution and downsampling are first performed to extract the high-level semantic features of the image, and then deconvolution is performed to upsample the features maps. The result of the deconvolution is spliced with the feature maps of the same size in the downsampling to obtain accurate pixel classes features information. The segmentation model we used is shown in Figure 1.


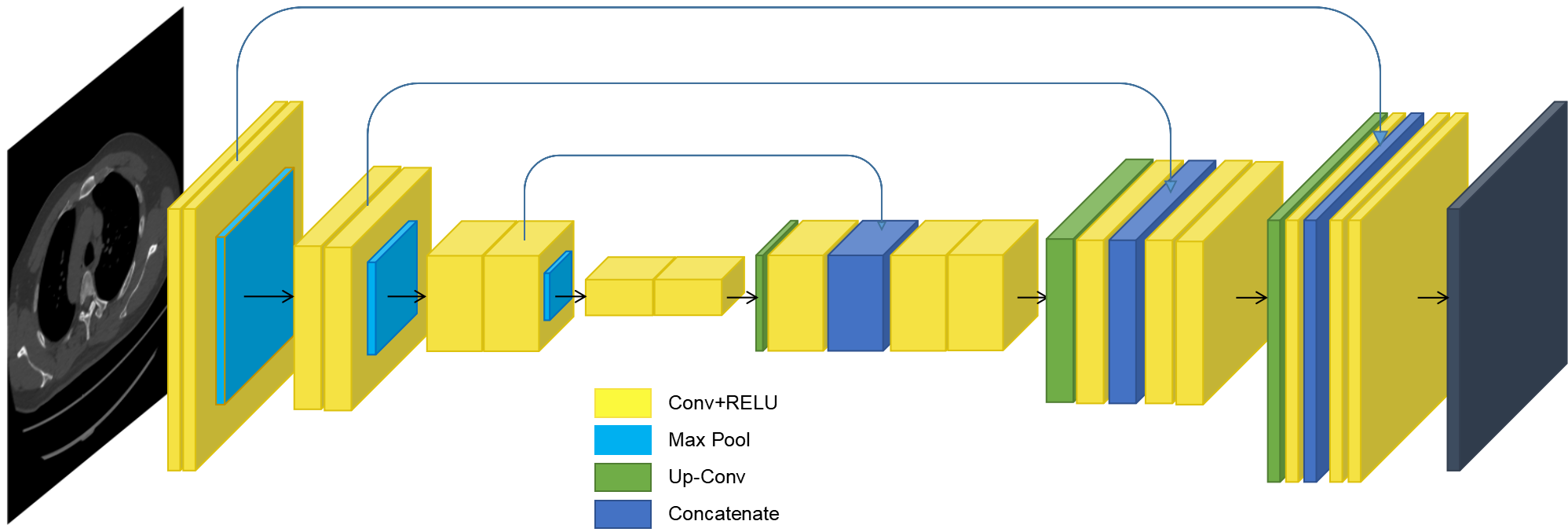


Figure 1. The architecture of bone segmentation.

**Rib locating**

To improve the recognition accuracy of the classification model of rib fracture, it is necessary to pick out the part of the rib. Because of the large proportion of the spine and scapulae in the chest bones, it is primary to focal on the removal of the spine and the scapulae.

Usually, the spine is located in the middle of the CT image and its thickness is greater than the thickness of the rib. According to this structural feature, each image in the CT sequence after bone segmentation is projected vertically, and then all projections are accumulated. By the above operations, a projection map can be obtained, and the horizontal coordinate corresponding to the highest peak point in the middle position of the projection map is the position of the centerline of the spine. After determining the position of the centerline of the spine, it is also necessary to confirm the left and right borders of the spine. Let x be the abscissa of the spine centerline, h be the highest projection value, then the abscissa of left border is the first position in region [x-50, x] whose projection value greater than h/2, the abscissa of right border is the first position in region [x, x+50] whose projection value less than h/2. Since the sternum is aligned with the spine in the vertical direction, the sternum can also be removed while removing the spine.

The scapulae also have some stable structural features. In the CT image, the scapula is located at the lower left (or leftmost) position and lower right (or rightmost) position, and there is a certain angle with the horizontal direction. The left ribs must be located at the upper right of the left scapula. The right ribs must be located at the upper left of the right scapula. The scapulae selection algorithm is as follows (only the left scapulae selection algorithm is described here, and the right scapula algorithm is similar to the left scapula).

Algorithm 1. The scapulae selection algorithm


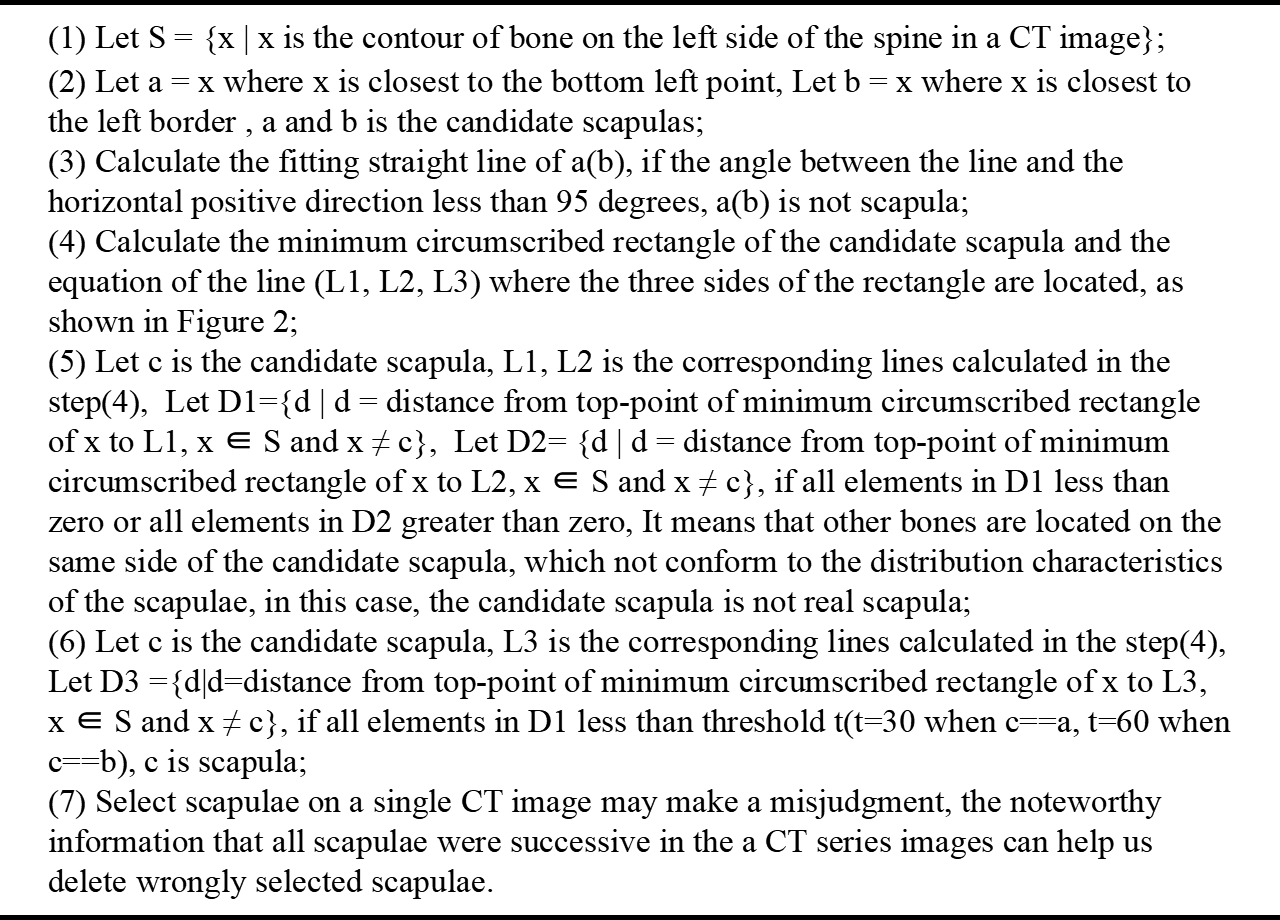


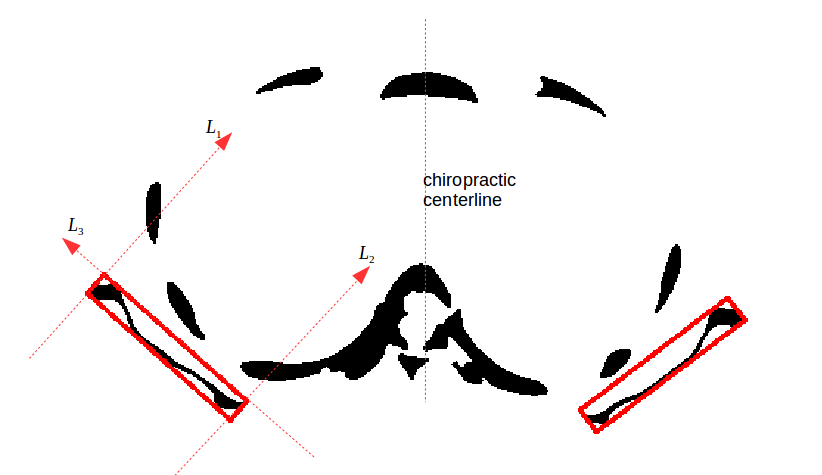


Figure 2. Schematic diagram of scapulae selection.

**Rib Fracture Classification**

DenseNet [3] is a densely connected convolutional network, recent work has shown that it performs well on classification and detection problems. The main idea of DenseNet is concatenating front and back layers in the network, this connection structure can alleviate the gradient dissipation problem in the training phase to a certain extent, and can also be of benefit to feature propagation and feature multiplexing. In addition, inspired by the design idea of Inception-v4 [4], we introduced the inception structure into Densenet to make the network applicable to different scales of receptive field. Otherwise, we extended 2D-CNN to 3D-CNN because of that 3D networks can capture more spatial stereo information around the bone block, make the model more reliable. The detailed model architecture of the rib fracture classification model was shown in Figure 3.


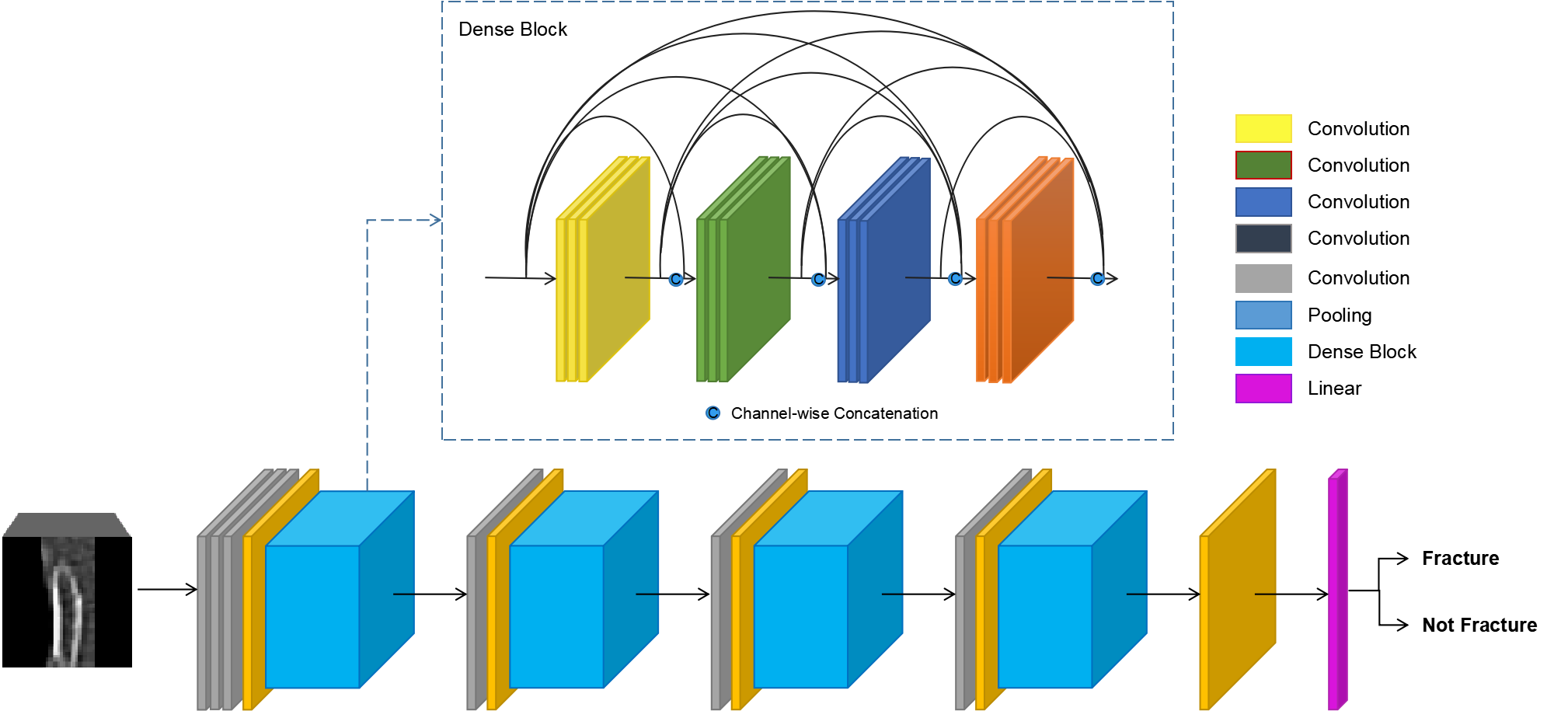


Figure 3. The architecture of rib fracture classification (3D DenseNet).

**The Discussion of Overfitting**

Data augmentation was used in the training of both U-Net and 3D DenseNet to avoid overfitting. We took the training of 3D DenseNet for example. The accuracy on training, validation, and test dataset with or without data augmentation are listed in Table 1. From Table 1, we can draw the conclusion that data augmentation can avoid the overfitting.

Table 1 The comparison of the accuracy on training, validation, and test dataset with or without data augmentation

| Data augmentation | Training | Validation | Testing |
| --- | --- | --- | --- |
| No | 0.986 | 0.827 | 0.816 |
| Yes | 0.976 | 0.923 | 0.931 |

**References**

1. Ronneberger O, Fischer P, Brox T. U-net: Convolutional networks for biomedical image segmentation. In International Conference on Medical image computing and computer-assisted intervention. pp. 234-241, 2015.
2. Long, J., Evan, S., and Trevor, D.. Fully convolutional networks for semantic segmentation. In Proceedings of the IEEE conference on computer vision and pattern recognition, pp. 3431-3440. 2015.
3. Huang, G., Liu, Z., Van Der Maaten, L. and Weinberger, K.Q. Densely connected convolutional networks. In Proceedings of the IEEE conference on computer vision and pattern recognition, pp. 4700-4708, 2017.
4. Szegedy, C., Ioffe, S., Vanhoucke, V. and Alemi, A. Inception-v4, inception-resnet and the impact of residual connections on learning. In Proceedings of the AAAI Conference on Artificial Intelligence，31(1)，2017.
